# Interdisk spacing effect on resonant properties of Ge disk lattices on Si substrates

**DOI:** 10.1038/s41598-022-11867-5

**Published:** 2022-05-17

**Authors:** A. A. Shklyaev, D. E. Utkin, A. V. Tsarev, S. A. Kuznetsov, K. V. Anikin, A. V. Latyshev

**Affiliations:** 1 Novosibirsk State University, 2 Pirogov Str., Novosibirsk, 630090 Russia; 2 Rzhanov Institute of Semiconductor Physics SB RAS, 13 Lavrentiev Aven., Novosibirsk, 630090 Russia; 3 Rzhanov Institute of Semiconductor Physics SB RAS, Novosibirsk Branch “TDIAM”, 2/1 Lavrentiev Aven., Novosibirsk, 630090 Russia

**Keywords:** Materials science, Nanoscience and technology, Optics and photonics

## Abstract

The light reflection properties of Ge disk lattices on Si substrates are studied as a function of the disk height and the gap width between disks. The interdisk spacing effect is observed even at such large gap widths as 500 nm. The gap width decrease leads to the appearance of the reflection minimum in the short wavelength region relative to one originated from the magnetic and electric dipole resonances in individual Ge disks, thereby essentially widening the antireflection properties. This minimum becomes significantly deeper at small gap widths. The observed behavior is associated with the features of the resonant fields around closely spaced disks according to numerical simulation data. The result shows the importance of using structures with geometrical parameters providing the short-wavelength minimum. This can essentially enhance their other resonant properties, which are widely used for applications, in particular, based on collective lattice resonances.

## Introduction

The efficiency of optoelectronic devices, such as solar cells and photodetectors, depends on their surface properties related to light reflection and transmission^1,2^. To reduce reflection, surface texturing and coating with antireflection dielectric films are usually used^3,4^. Recently, intensive research has been carried out on coatings of metal and/or dielectric particles as an alternative to continuous antireflection films^5–9^. Their action is based on the excitation of plasmonic^10,11^ or electromagnetic (EM) resonances^12–15^ in metal and dielectric particles, respectively. Compared to continuous films, the capabilities of antireflection coatings made of particles are wider. They can be less dependent on the EM radiation incidence angle^5^. In addition, they can be more broadband^5,9^, or, on the contrary, have a greater spectral selectivity^6,8,16,17^ due to using various resonance effects.

The characteristics of optical resonances are well studied for individual dielectric particles, while their arrays are used for practical applications. An experimental study of metal particle arrays revealed the effects of collective lattice resonances which provide laser generation^17,18^, biosensing applications^19,20^ and amplification of light radiation^21–23^. In addition to creating antireflection coatings, arrays of ordered dielectric particles can be used in optical waveguides^24,25^ and also as metasurfaces and metamaterials^23,26^. Particular attention is paid to the study of the interaction of resonant modes, excited in individual dielectric particles, with lattice modes. In this case, it is reasonable to expect that the role of lattice modes will increase with a decrease in the interparticle spacing as a result of an increase in the overlap of their fields and the appearance of diffraction effects^27–29^.

The comparison of light scattering efficiencies of metal (Ag, Au) and dielectric (Si) particles showed that metal particles are more efficient at their small sizes (less than 100 nm) and, accordingly, in the wavelength region below 500 nm, whereas dielectric particles scatter stronger at their larger sizes in the region of longer wavelengths^30^. As for the comparison of coatings made of them, Si particle arrays on Si substrates provide a stronger light absorption, but in a narrower spectral region than Ag particle arrays^7^.

The EM radiation scattering by dielectric particles depends on their shape. In case of spherical and cubic particles, which are characterized by only one geometrical parameter, magnetic and electric dipole resonances are located at different wavelengths, and their mutual positions do not change when the particle size varies^12,31^. At the same time, for particles described by two geometrical parameters, e.g., diameter *d* and height *h*, as for disk-shaped particles, the spectral position of these resonances relative to each other depends on the aspect ratio (AR) determined as AR = *h*/*d*. Calculations have shown that wavelengths of the resonances coincide at AR ≈ 0.76^31^ for Si disks in the air. The AR value at which they coincide differs from that for disks on substrates depending on their optical properties^8,14,32,33^. When the scattering properties of magnetic and electric dipole modes are balanced, their interference with the incident EM radiation most efficiently suppresses both reflected and transmitted waves^7,33^. Due to such destructive interference, the resulting EM radiation propagates in the substrate surface layer plane, which is important for the efficient operation of optoelectronic converters.

Another parameter affecting the light reflection and transmission by arrays of dielectric particles is the gap width (*G*) between them, on which the interaction between resonant EM fields strongly depends. The study of the *G*-related effect was carried out for dimer-forming particles^34,35^ and structures of several particles with different configurations^36^. The strongest interaction was observed in cases of collective lattice resonances^8,27,37,38^, which are well studied for plasmon resonances^17,37^. In case of dielectric particles, the collective lattice resonances are usually studied for structures with rather large *G* values. Nevertheless, the interaction of resonant modes at small G deserves a careful study.

In this work, we experimentally studied the effect of the interparticle spacing value in regular arrays on their reflection spectra. The particle coatings were shaped as a square lattice of Ge disks with a diameter of about 200 nm on Si substrates. It is found that the influence of the *G* value on the light reflection is stronger than the effect of such parameter as AR, which usually dominates when EM radiation is scattered by disk-shaped particles. Moreover, at small *G* values, a deep reflection minimum is formed at wavelengths shorter than the spectral position of the minimum originated from magnetic and electric dipole resonances generated in individual dielectric particles. The conditions for this minimum appear to be more suitable for using resonant effects for various applications, in particular, based on collective lattice resonances.

## Experimental results and discussion

**Figure 1:**
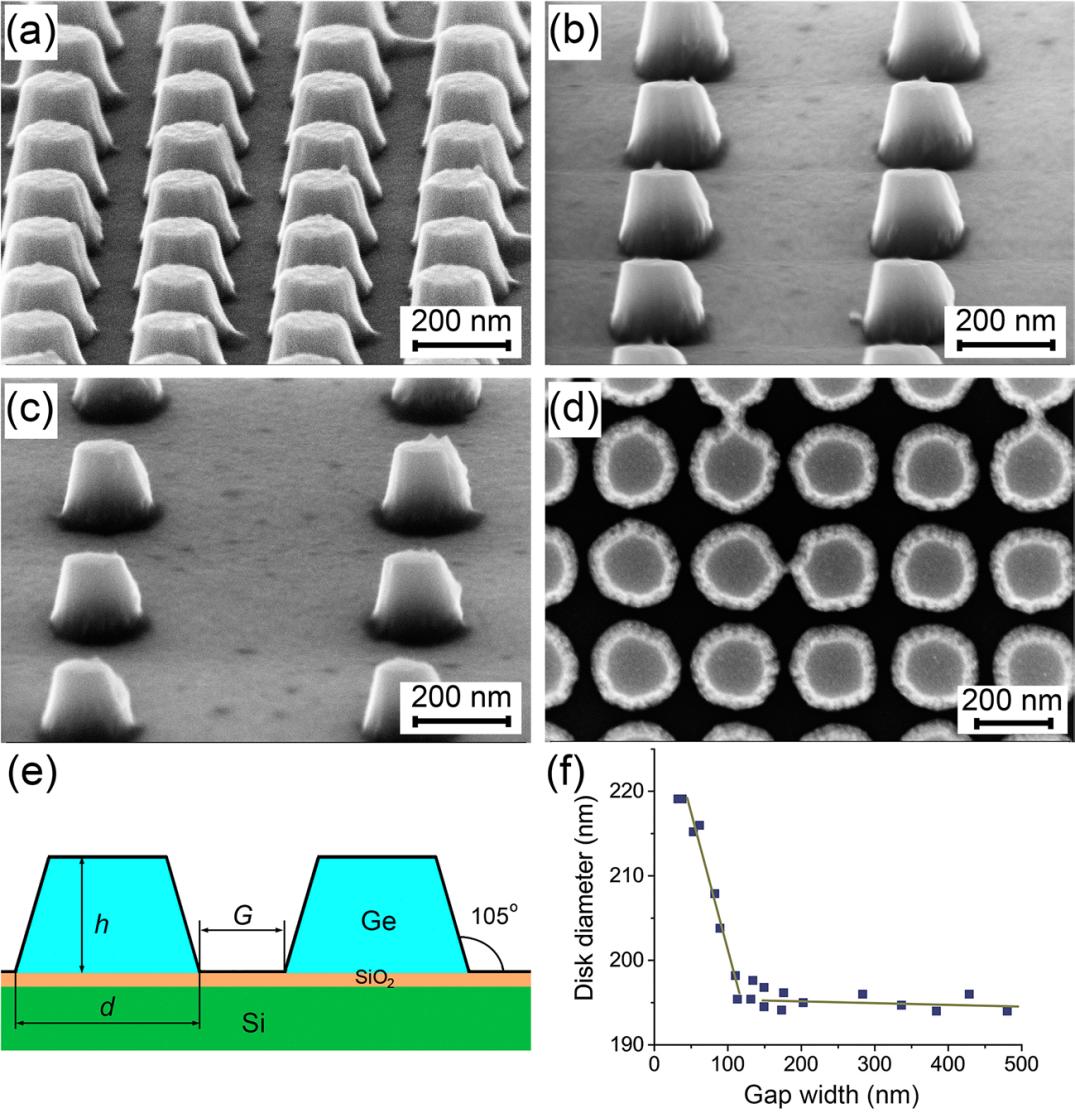
(**a**–**c**) SEM images of Ge disks with diameters of about 200 nm at their base and a height of about 136 nm. The distance between the disk centers was 300, 500 and 700 nm in (**a–c**), respectively. The images are taken at the sample inclination angle of 45°. (**d**) SEM image of close-spaced Ge disks with a height of 90 nm obtained at a normal e-beam incidence angle. (**e**) Schematic cross-section of the structure. (**f**) Dependence of the disk diameter at their base on the *G* value.

The method we use leads to fabricating particles which shape is not perfectly cylindrical (Fig. 1). The particle sidewalls are tilted about 15° relative to the vertical^14^, and the particles are shaped as truncated cones, as schematically shown in Fig. 1e. Since the tilting angle is small, hereinafter, we will call them disks. The disk tilting angle is determined by the sidewall inclination of the opened windows in the electronic resist^14^. Another feature of the resist electron beam exposure is that the resulting disk diameter is somewhat larger when the disks are located close to each other (Fig. 1f). This occurs when the G value becomes less than 100 nm. This feature is less pronounced on the average disk diameter and consists more in the change of the disks shape. There is a slight disk shape deviation from the truncated cone through a diameter increase at its base (Fig. 1a). The data points in Fig. 1f show the spread of disk diameters within each disk array, which is less than 1%. It can also be noted that the smallest reproducible gap width was about 50 nm. When trying to get disks separated by a smaller *G*, bridges appeared between the disks, as shown in Fig. 1d. Deposited Ge is polycrystalline^14^, consisting of clusters with a lateral size of up to 25 nm. The polycrystallinity of structure of the Ge disks is displayed as graininess in their surface images.

**Figure 2:**
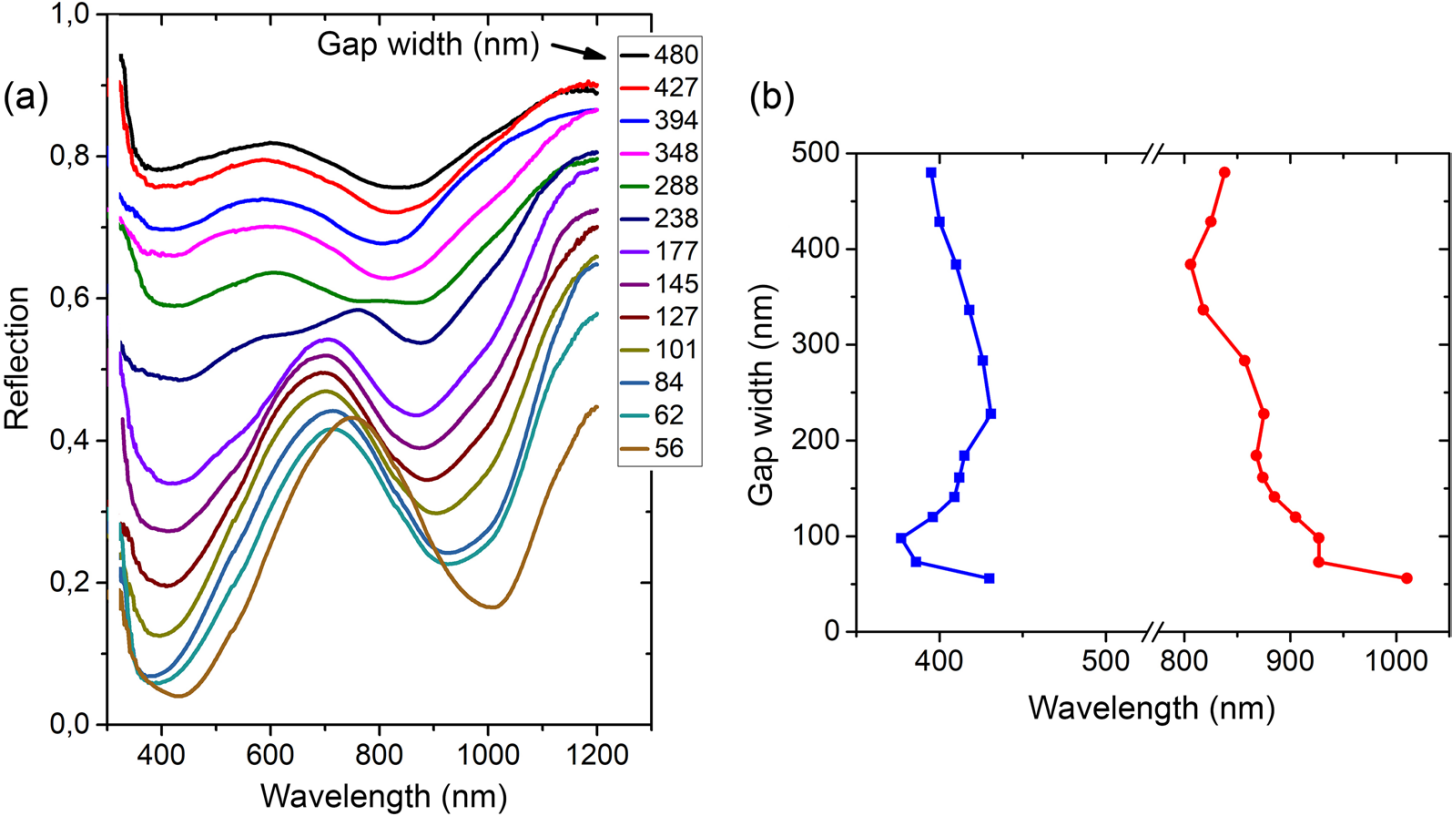
(**a**) Reflection spectra of Ge disk coatings with the disk height of 120 nm. The gap width values between the disks at their base are marked in the figure. (**b**) Spectral position of the two minima versus the *G* value for coatings with 120 nm high disks. The accuracy of determining the position of the minima at large *G* values is limited by their flat shape and was within ± 5 nm.

The reflection spectra of Si substrates with the Ge disk coatings contain two minima which exhibit a strong dependence on *G* (Fig. 2a). The spectral positions of the minima as a function of *G* is rather complicated (Fig. 2b). The shifts of the minima to shorter wavelengths at small *G* value (~ 50 nm) can occur due to the larger average diameter of the corresponding disks (Fig. 1f). The reflection minima for coatings with large *G* values are rather flat. This limits the accuracy of determining their spectral position.

**Figure 3:**
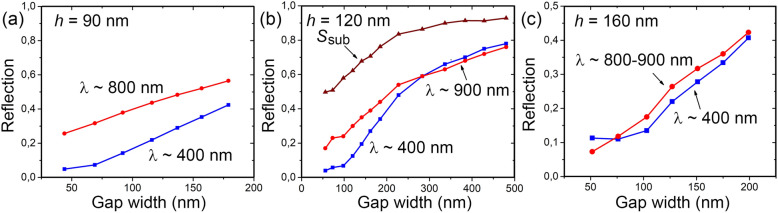
(**a–c**) Dependences of the reflection level in two minima at wavelengths ~ 400 and 800–900 nm on the *G* value for arrays with disks 90, 120 160 nm high, respectively. S_sub_ in (**b**) is the fraction of the surface not occupied by Ge disks.

A decrease of the *G* value is accompanied by an increase of the disk concentration on the surface. This leads to a decrease in the reflection level in the entire investigated spectral region (Fig. 2a). It is shown in Fig. 3 how this decrease occurs for the two reflection minima located near 400 and 800–900 nm. For 120 nm high disks (Fig. 3b), these data were obtained in a wide range of *G* values. The comparison of the surface filling factor by the disks (S_sub_) with the reflection level in the minima shows that this decrease occurs faster than an increase in the disk concentration. At the same time, the reflection in these minima decreases differently upon *G* so that the reflection in the region of 400 nm has a stronger dependence than that in the region of 800–900 nm.

The stronger decrease in reflection as a function of decreasing *G* value in comparison with decreasing the surface area not covered with the disks, as shown in Fig. 3b, in the whole range of *G* values studied here, indicates the presence of the coupling effect for the resonant magnetic and electric moments of the disks even at such large inter-disk distances as 500 nm. For the array of Ge disks with a diameter of about 200 nm, the magnetic and electric dipole resonances are located in the region of 800–1000 nm, depending on the disk AR value^14,31,32^. The reflection minimum is observed when magnetic and electric resonances are balanced^7,39^ and, therefore, the reflection minimum in this spectral region is due to dipole resonances. The reflection minimum at wavelengths near 400 nm can be associated with the resonances involving quadrupole modes, the field of which is more localized around the disks compared to dipole modes. This difference in the resonance field localization can be the reason for the stronger reflection dependence on *G* at these wavelengths.

**Figure 4:**
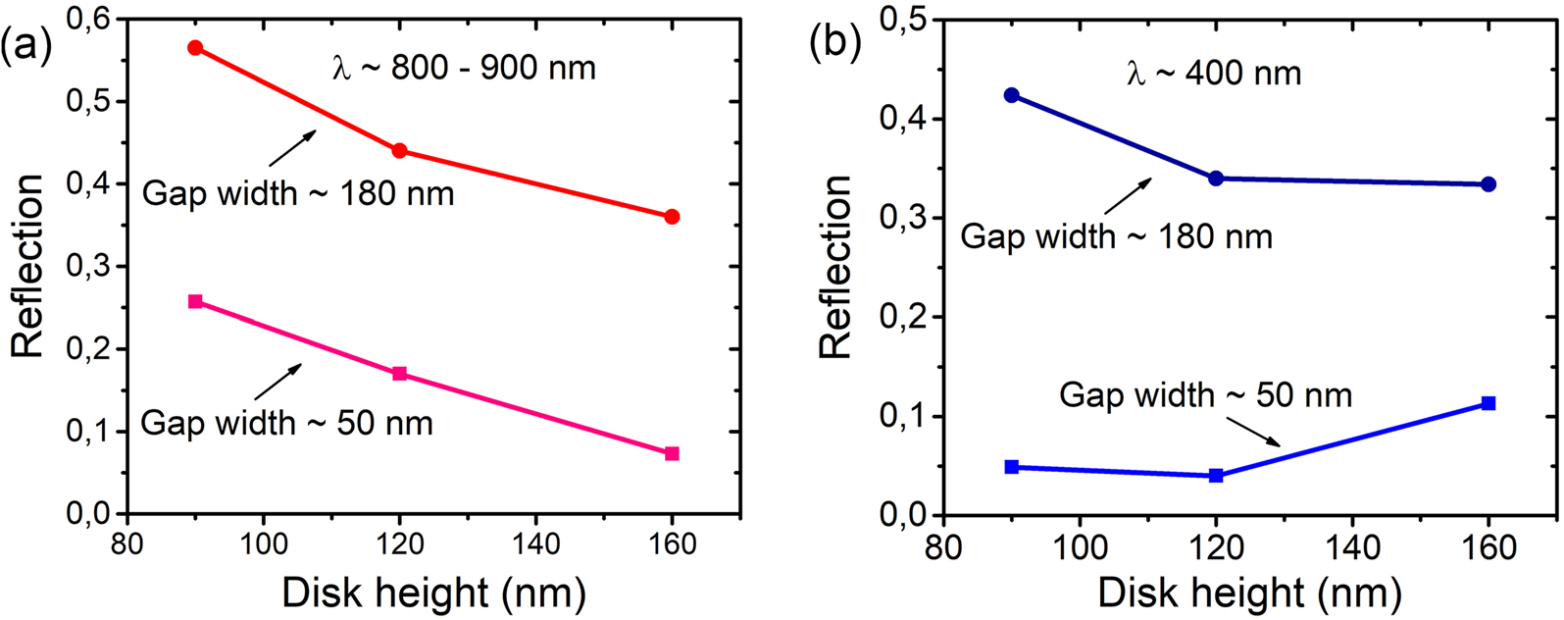
Reflection level in two minima at wavelengths (**a**) 800–900 and (**b**) ~ 400 nm as a function of the disk height for two *G* values of about 50 and 180 nm.

The reflection minimum depth at 800–900 nm is significantly decreased with increasing the disk height, as shown in Fig. 4a for small (~ 50 nm) and relatively large (~ 180 nm) *G* values. This behavior is consistent with the previously obtained results^14,31^ and associated with the AR dependence of spectral positions of magnetic and electric dipole resonances. Their positions approach each other with an increase in AR to ~ 0.8^14,31^, which enhances the conditions arising at larger amplitudes of magnetic and electric dipole resonance modes. There is a factor that can reduce the scattering properties of Ge particles on Si. This is the resonance EM field leakage into the substrate, which was observed for SiGe particles^40,41^. This effect is stronger for particles with lower AR values^9^.

For the reflection minimum at wavelengths near 400 nm, the different dependence on AR is observed for different G values (Fig. 4b). Here, at low *G* values (~ 50 nm), the reflection minimum is even deeper for disks with relatively low AR values (< 0.5). This difference in the behavior of reflection minima as a function of AR indicates that the lattice effect is stronger in the region of shorter wavelengths. This occurs despite the fact that the reflection minimum in the region of longer wavelengths is determined by the magnetic and electric dipole resonance modes.

## Numerical simulation

**Figure 5:**
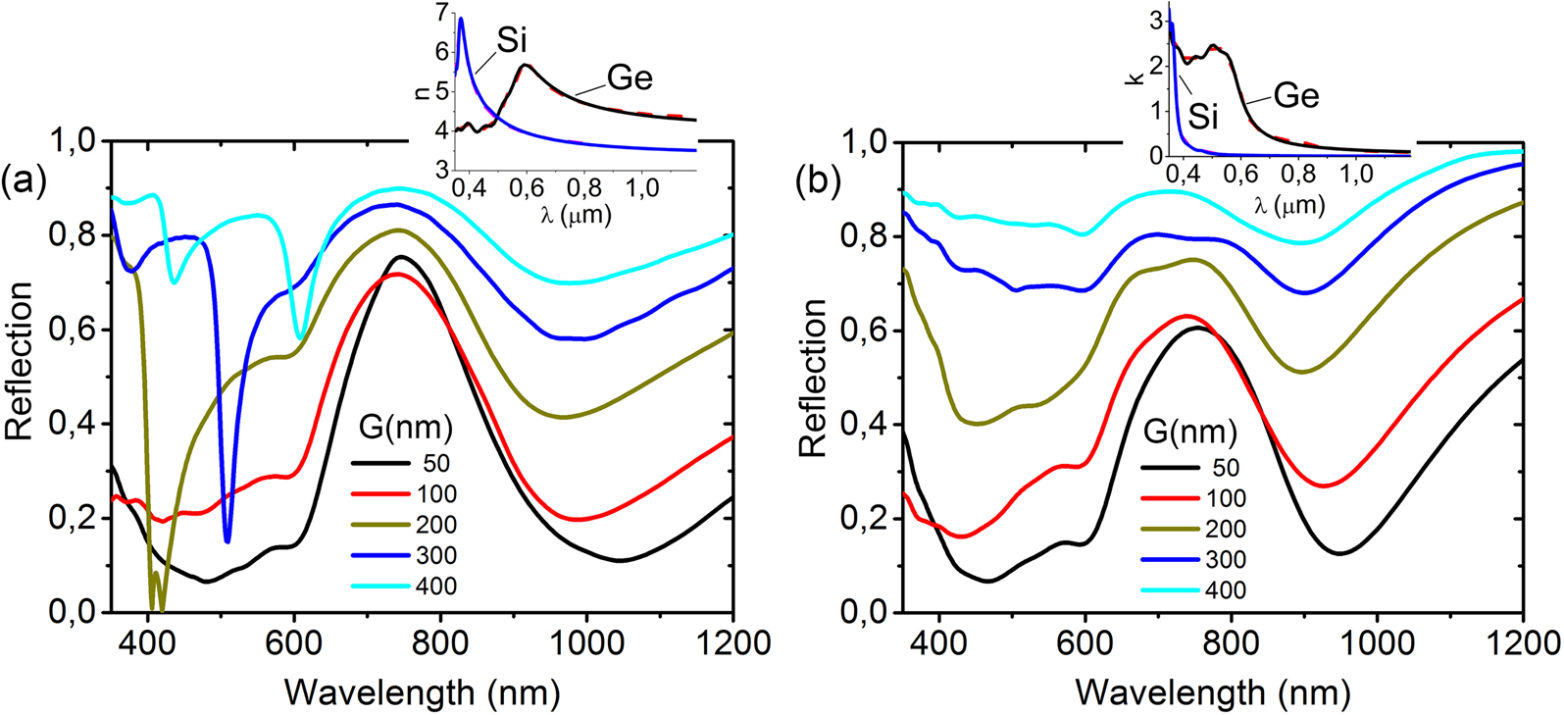
Normalized reflectance for arrays with different numbers of disks: (**a**) infinite, (**b**) 21 × 21. The structure was irradiated with a plane wave in (**a**) and with a highly divergent Gaussian beam, which can be formed by an objective with NA = 0.65 in (**b**). The disks were 200 nm in diameter and 120 nm high. The *G* values are marked in the figures. The dispersions of *n* and *k* for Si and Ge used in the simulation are shown in the inserts in (**a,b**), respectively.

The electromagnetic simulations of Ge disk arrays on Si substrates in the configuration of Fig. 1e were performed with the finite difference time domain (FDTD) method using the commercial FullWave software package from RSoft-SYNOPSYS^42^. The material dispersion in FDTD simulations in general requires fitting an actual dispersion to a simple model as a series of Lorentzian expressions^43^. We used the built-in material editor that uses the known experimental data for Si, Ge and SiO_2_^44^. In our case, the fitting in the wavelength range from 0.35 to 1.2 µm was carried out with a moderate number of Lorentz nodes (up to 6) to ensure a good fit to the experimental data of the real (*n*) and imaginary (*k*) parts of the refractive indexes (plots of the data used for Si and Ge are shown in the inserts in Fig. 5).

Typically, periodic structures are simulated by using the unit sell that contains a single disk with periodic boundary conditions in the structure plate and a perfectly matched layer (PML) with an absorbing boundary condition^43^ in the normal direction. The calculated reflection spectra for our case are shown in Fig. 5a for different gaps and a fixed disk diameter and height. Similar to experiments, the calculated spectra for the Ge disk arrays are normalized to the bare substrate spectra. It can be seen that the experimental and calculated spectra agree well at small G values and differ strongly at large G values. These discrepancies are due to the fact that this simulation corresponds to an infinitive periodic structure and a plane incident wave. However, the experimental setup uses a strongly divergent optical wave formed by an objective with a big NA = 0.65.

In order to simulate the conditions of the experimental setup, we use the cell with a large number of disks (21 × 21 = 441) and PML boundary conditions on all sides. This drastically increases the calculation time, but gives the results (see Fig. 5b) that quantitatively agree to our experiments. We also calculated the spectra for a low divergent incident wave (corresponding to an objective with NA = 0.1), which were very similar to the spectra (Fig. 5a) calculated for the infinite structure irradiated by a plane wave. This means that, when using an objective with a small NA, an additional minimum should appear in the reflection spectra for structures like ours with relatively large G values.

Since, according to the previous studies, the origin of the minimum in the long-wavelength region of the reflection spectra is associated with the excitation of magnetic and electric dipole modes^12,14,31,32^, the main goal of our numerical studies is to clarify the origin of the reflection minimum in the short wavelength region around 400 nm. Just like in the experiments, two minima are observed in the calculated reflection spectra in the region of 350–1200 nm (Fig. 5). The comparison of Fig. 5a,b shows that the depth of the minimum in the long-wavelength region of the reflection spectra is practically the same for the structures with the same *G* value, i.e., it does not depend on the incident wave divergence, as well as on the number of disks. At the same time, the incident wave divergence strongly affects the width of the minimum and its depth at large G values in the short wavelength region of the reflection spectra.

**Figure 6:**
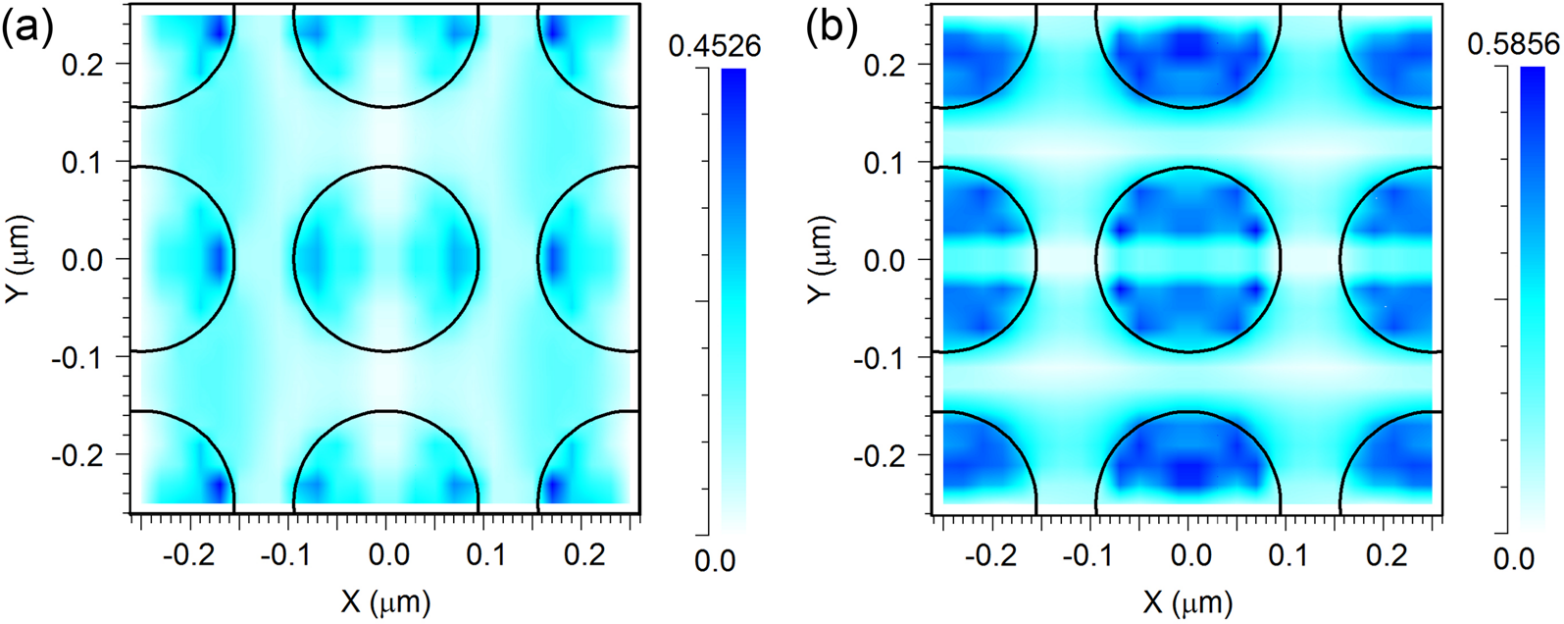
Lateral distribution for the electric (**a**) and magnetic (**b**) field components at the wavelength of 598 nm in the cross-section Z = 20 nm for *G* = 50 nm.

**Figure 7:**
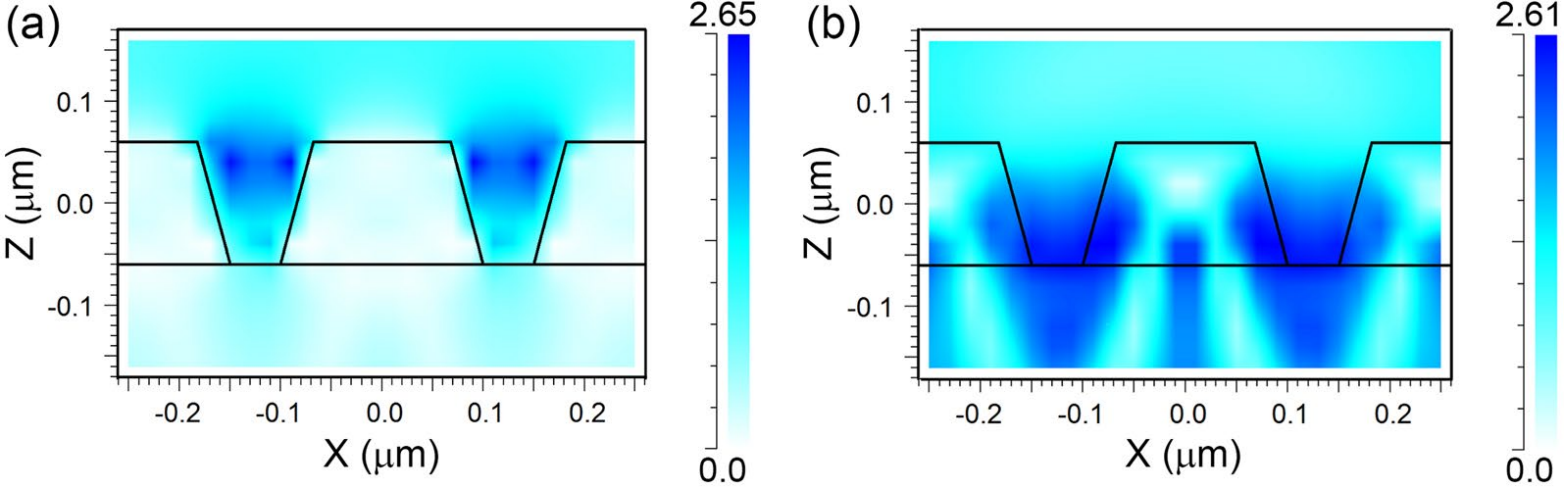
Lateral distribution for the electric (**a**) and magnetic (**b**) field components at the wavelength of 598 nm in the cross-section Y = 0 for *G* = 50 nm.

To clarify the origin of the resonance in the arrays under study, the spatial distribution of the electric and magnetic fields in the disk area for two wavelengths in the region of minima in the reflection spectra were calculated. The exciting wave was considered to be normally incident upon the substrate surface with polarization along the X axis. For the reflection minimum in the long-wavelength region of the spectrum, it was found that the resonant field maximum is located inside the disk with the predominance of magnetic dipole mode (*H*_y_). For the reflection minimum in the short-wavelength region, the field distribution in the disk area corresponds to the electric dipole and magnetic quadrupole modes (Fig. 6). In addition, the field maximum is concentrated in the space between the disks (Fig. 7). The maximum field amplitudes turn out to be greater than those of the fields in the long-wavelength region of the spectrum. Due to these factors, the minimum reflection in the short-wavelength region becomes deeper.

The collective lattice resonances are practically not observed in the calculated spectra (Fig. 5b) in the case of a 21 × 21 disk array for the strongly divergent optical wave. These resonances arise (Fig. 5a) when a plane wave is incident on an infinite lattice of disks in air (*n* = 1) as well as at the incidence of a low divergent wave on the 21 × 21 disk array. The spectrum of the lattice with *G* = 50 nm does not contain a lattice resonance peak, since it is located in the shorter wavelength region. In this case, the calculated and experimental spectra are in a good agreement with each other. The resonance minima in the calculated spectra appear at large *G* values (Fig. 5a). It can be noted that the resonance minima are not narrow. This is due to the influence of the Si substrate, the refractive index of which significantly differs from that of the air surrounding the disks. As shown in^45^, in such cases, the resonance Q-factor is decreased. The low Q-factor of the structure of Ge disks on a Si substrate is also associated with a small difference in the refractive indices between the materials of the disks (Ge) and the substrate (Si), which causes the resonant mode field to leak into the substrate^9^.

In our experiment, a wide non-monochromatic EM radiation beam from a halogen lamp passed through the microscope objective with NA = 0.65 and is focused on the area with a diameter of about 80 μm. As a result, EM radiation falls onto the sample at different angles, which cover the range up to 20° (half of the objective aperture angle). It has been shown that the spectral position of the lattice resonance depends on the EM beam incidence angle^28^. Our numerical simulations show that the contributions from partial waves impinging at angles greater than 7° completely smooth out the effect of the collective lattice resonances, thus, making them invisible in our experiments. In addition, as the angle of incidence deviates from the normal one, the resonance Q-factor is decreased^37^. Despite the absence of narrow minima due to collective lattice resonances, their effect, nevertheless, manifests itself in a greater depth of the efficient broad minimum in the short-wavelength region of our spectra. This agrees to the results of^38^, which showed that the scattering maxima caused by lattice resonances can be located far from the strongest EM resonances of individual particles, which, in our case, are of the dipole type located in the region of relatively longer wavelengths.

## Conclusion

In this study we found that the influence of the close spacing of dielectric disks in their lattices on the reflection spectra is observed even at such large gap widths between the disks as 500 nm. The gap width value effect turns out to be stronger in the short-wavelength region of the measured spectra. According to our numerical simulations, this is due to the participation of quadrupole resonances and stronger resonant fields in the disk area caused by the close proximity of the disks. As a result, at small gap widths, the appearing reflection minimum in the short-wavelength region of the spectra is essentially deeper than the minimum in their long-wavelength region, which originated from magnetic and electric dipole resonances in individual dielectric disks. The stronger gap width value that influences the reflection in the short-wavelength region should be taken into account both in the manufacture of antireflection coatings and sensors which operation is based on the use of local resonant EM fields. As for collective lattice resonances designed for the conditions of the short-wavelength reflection minimum, they can be more efficient in their various applications, compared to those created for the conditions of magnetic and electric dipole resonances.

## Methods

### Ge disk fabrication

Ge disk arrays were fabricated on Si substrates coated with a 5 nm thick thermal SiO_2_ film, similar to how it was made in^14^. After a positive resist PMMA 950 K A4 film deposition on the substrate, it was exposed to a 20 keV electron beam at the aperture of 10 μm using the Raith PIONEER lithography system. Circle-shaped holes were formed by selective dissolving the PMMA films in the methyl isobutyl ketone and isopropyl alcohol (IPA) solution taken as 1:3, respectively, at room temperature for 30 s. The fabricated masks consisted of holes of about 200 nm in diameter arranged in a square lattice. The distance between hole centers was varied from about 250 to 700 nm. Ge films of different thicknesses were deposited onto the prepared samples by the Ge evaporation from a Knudsen cell in an Omicron ultrahigh vacuum system. To obtain Ge disk arrays, excess Ge was removed from the sample surface through the lift-off process in an ultrasonic bath with acetone for 1 min.

### Methods of characterization

The shape of Ge disks after their formation and the distance between them were determined using the PIONEER lithography system (in the microscope mode) or a scanning electron microscope (SEM) manufactured by Hitachi (SU 8020). The reflection spectra at a normal light incidence were measured with the microscope-spectrophotometer MSFU-K supplied with a 40 × objective (WD = 0.6 mm, NA = 0.65). This optical setup collects reflected light within the objective aperture angle α = 40.5°. The sample position was shifted about 0.2 mm relative to the objective focal plane for the irradiation of a wide (~ 80 μm) area containing a large number of Ge disks. The reflection spectra for Ge disk arrays were normalized to those of bare substrates.

## Data Availability

The data that support the findings of this study are available from the corresponding author upon reasonable request.
